# An aerosol deposition based MEMS piezoelectric accelerometer for low noise measurement

**DOI:** 10.1038/s41378-023-00484-5

**Published:** 2023-03-06

**Authors:** Xuewen Gong, Yu-Chun Kuo, Guodong Zhou, Wen-Jong Wu, Wei-Hsin Liao

**Affiliations:** 1 Department of Mechanical and Automation Engineering, The Chinese University of Hong Kong, Hong Kong, China; 2 Department of Engineering Science & Ocean Engineering, National Taiwan University, Taipei, Taiwan; 3 Department of Electronic Engineering, The Chinese University of Hong Kong, Hong Kong, China

**Keywords:** Electrical and electronic engineering, Materials science

## Abstract

Potentially applied in low-noise applications such as structural health monitoring (SHM), a 1-axis piezoelectric MEMS accelerometer based on aerosol deposition is designed, fabricated, simulated, and measured in this study. It is a cantilever beam structure with a tip proof mass and PZT sensing layer. To figure out whether the design is suitable for SHM, working bandwidth and noise level are obtained via simulation. For the first time, we use aerosol deposition method to deposit thick PZT film during the fabrication process to achieve high sensitivity. In performance measurement, we obtain the charge sensitivity, natural frequency, working bandwidth and noise equivalent acceleration of 22.74 pC/g, 867.4 Hz, 10–200 Hz (within ±5% deviation) and 5.6 $\mu {{{\rm{g}}}}/\sqrt{{{{\rm{Hz}}}}}$ (at 20 Hz). To demonstrate its feasibility for real applications, vibrations of a fan are measured by our designed sensor and a commercial piezoelectric accelerometer, and the results match well with each other. Moreover, shaker vibration measurement with ADXL1001 indicates that the fabricated sensor has a much lower noise level. In the end, we show that our designed accelerometer has good performance compared to piezoelectric MEMS accelerometers in relevant studies and great potential for low-noise applications compared to low-noise capacitive MEMS accelerometers.

## Introduction

Vibration measurement is required in a quantity of applications, such as structural health monitoring^1^, machine condition-based monitoring^2^, inertial navigation^3^, earthquake early warning^4^, health monitoring of patients^5^, etc. To detect vibration signals in these applications, low noise accelerometers are generally used. Traditional piezoelectric accelerometers already have superior low-noise performance to meet the demand, such as MMF KB12VD and B*&*K Type 8344-B-002. However, the market still looks for lighter and cheaper choices. The evolution of microelectromechanical systems (MEMS) accelerated the miniaturization and cost reduction of all kinds of devices^6–9^, enabling low noise capacitive MEMS accelerometer to be developed and applied to these applications. Commercially available products such as SI1003 by Colibrys and ADXL1001 by Analog Devices have already achieved in-band noise densities from sub - $\mu {{{\rm{g}}}}/\sqrt{{{{\rm{Hz}}}}}$ to dozens of $\mu {{{\rm{g}}}}/\sqrt{{{{\rm{Hz}}}}}$. Reported papers in recent years have shown even lower noise levels^10–13^. However, to reduce noise, capacitive MEMS accelerometers usually adopt vacuum packaging to achieve high quality factor, which might cause additional circuit noise, higher cost, and fabrication complexity^14^. In some scenarios with frequent temperature change, they are also subject to the influences of temperature so that inducing thermal drift, characterized by bias drift and scale factor drift^15,16^. By contrast, piezoelectric accelerometers have high quality factors, which reduce the need for vacuum encapsulation. Together with good temperature durability and linearity^17^, they would be reliable choices in many industrial applications. Keeping in mind these advantages, we see using the microfabrication process to develop piezoelectric MEMS accelerometers as a great opportunity as it could provide low-noise accelerometers with smaller dimensions to fulfill the market expectation.

For achieving piezoelectric MEMS accelerometers, cantilever beam and symmetric suspension structures are mainly adopted as they lead to higher sensitivities on small scale^18^. As for commonly used sensing materials, aluminum nitride (AlN), lead zirconate titanate (PZT), and zinc oxide (ZnO), AlN has a lower dielectric constant and better compatibility with CMOS fabrication lines. In contrast, PZT has the advantage of much higher piezoelectric constants compared to AlN and ZnO^19^. To deposit PZT layer for accelerometers, sol-gel method^17^ and magnetron sputtering^20^ have been used to fabricate around 2 μm thick film. Combinations of these structures, materials, and deposition methods have been explored to fabricate piezoelectric accelerometers. Wang et al. fabricated accelerometers based on sol–gel deposited PZT films with annular diaphragm structures. They had measured sensitivities from 0.77 to 7.6 pC/g and resonant frequencies from 35.3 to 3.7 kHz^21^. Hewa–Kasakarage et al. developed PZT cantilever accelerometers using sol–gel deposition, achieving 3.4–50 pC/g charge sensitivities and 60–1.5 kHz resonance frequencies^17^. Saayujya et al. also made an accelerometer with the same structure type using ZnO material. Voltage sensitivity of 1.69 mV/g and natural frequency of 2.19 kHz were realized in this case^22^. Using AlN, Gesing et al. fabricated a structure composed of four-symmetric suspension beams with a 510 $\mu {{{\rm{g}}}}/\sqrt{{{{\rm{Hz}}}}}$ noise density, 0.0981 pC/g charge sensitivity and 19.1 kHz natural frequency^23^. Trivedi et al. also demonstrated a four-symmetric structure. They applied 1 μm PZT layer and obtained the noise density, sensitivity and natural frequency of 5800 $\mu {{{\rm{g}}}}/\sqrt{{{{\rm{Hz}}}}}$, 8.12 mV/g, 9.62 kHz, respectively^24^.

None of the studies has attempted to apply PZT thick film deposition (tens of μm) on micro piezoelectric accelerometers, even though it is highly desirable. For either cantilever or suspension beam structure, the beam parts act as the function of spring, which means thinner beams increase the sensitivity of the sensor^14^, yet they should be kept above a certain thickness, e.g., tens of μm, so as to maintain mechanical strength of the structure. Due to this constraint, thicker piezoelectric film means a higher volume ratio of sensing material, leading to higher electrical output under the same size when being excited by accelerations, namely, higher sensitivity of the sensor. However, the commonly used sol–gel deposition is undesirable for achieving thick film, which is the process of a series of steps, including spin-coating of chemical solution of PZT precursor complexes, drying, pyrolysis, and sintering^25^. It facilitates the fabrication of high-quality and large-scale oxide films^26^. Nevertheless, the film is prone to crack during multiple rounds of heat treatment due to material shrinkage and the different thermal expansion coefficients of the PZT layer and the substrate. The risk of cracking would be higher when the thickness of the deposited film increases. This is where the aerosol deposition method plays a role. The method was applied in our previous study to fabricate accelerometers^27^. It is a process of forming a dense, uniform and hard ceramic layer in tens μm at room temperature when sub-micron ceramics particles are accelerated and impacted on a substrate by gas flow^28^. Compared to other fabrication methods such as sol-gel and sputtering, it is easy to pattern, has a low process temperature, and can be used to fabricate PZT film at a much quicker speed with excellent quality. These characteristics are of vital importance for the fabrication of microelectromechanical systems^29–31^.

In this paper, we still use aerosol-deposition-based PZT to fabricate a cantilever beam MEMS accelerometer and explore its potential for low noise applications. We first introduce the design of the structure with *d*_31_ mode sensing principle. Simulation is performed to obtain the simulated specification of the sensor, including sensitivity, natural frequency, more importantly, to validate whether it has a proper working range and noise level. Next, the measured specification of the accelerometer is presented, including natural frequency, frequency response, charge sensitivity, and noise density. To prove its usage in real applications, we measure the vibrations of a fan with the designed sensor, using a traditional piezoelectric accelerometer as a reference. Besides, its noise floor is compared with a low noise capacitive MEMS accelerometer. We then discuss its performance with a list of piezoelectric MEMS accelerometers in recent studies and commercially available low-noise MEMS accelerometer products. Finally, we introduce the fabrication process based on aerosol deposition, demonstrate the fabricated sensor, and present the experimental setup for measurements.

## Results

### Design and simulation

**Fig. 1:**
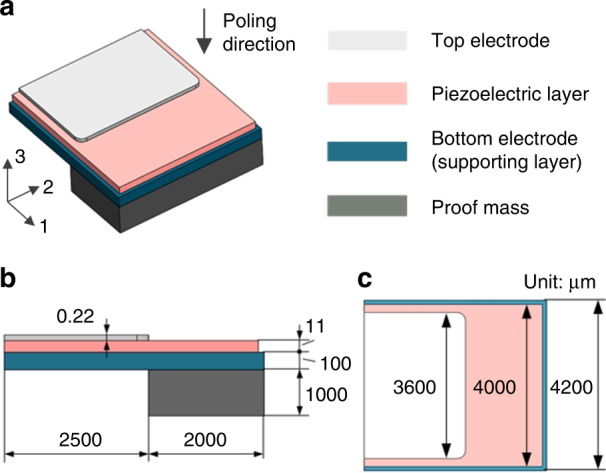
Structure design of the 1-axis accelerometer. **a** Structure and layer materials, **b** side view, **c** top view of the accelerometer

The 1-axis accelerometer is a cantilever beam structure, as shown in Fig. 1a. It has four functional layers: a top electrode, a piezoelectric sensing layer, a bottom electrode, and a proof mass. Accordingly, the materials to form the four layers are Pt/Ti, PZT, stainless steel, and tungsten. The side view of the accelerometer is presented in Fig. 1b. The left side is the fixed end of the beam, while the right side is the free end attached with a proof mass, which can be used to tune the sensitivity of the accelerometer. In the middle, the piezoelectric sensing layer and bottom electrode form the composite cantilever beam. Compared to a fully clamped centrosymmetric structure, a cantilever beam provides higher sensitivity under the same size due to lower beam stiffness. Besides, the design of a stainless-steel-based bottom electrode simplifies the fabrication process as it exempts the deposition of another layer of electrode^18^.

The top view of the accelerometer is shown in Fig. 1c. The piezoelectric sensing layer is slightly smaller than the bottom electrode. This design can protect the piezoelectric sensing layer during the wet etching process. The top electrode is of smaller size to prevent possible short-circuit leakage with the bottom electrode.

Directions in the piezoelectric element are denoted by 1–3 axes as shown in Fig. 1a. Aligned with the opposite direction of the 3 axis, the poling direction of the piezoelectric materials is perpendicular to the top surface of the PZT layer. The piezoelectric beam works in *d*_31_ mode. This is because when the accelerometer is excited by an acceleration in the 3 direction (vertical direction), the proof mass will move in this direction. The motion of the proof mass will induce the strain of the piezoelectric element in the 1 direction, therefore making the charges to accumulate on electrodes in the 3 direction.

**Fig. 2:**
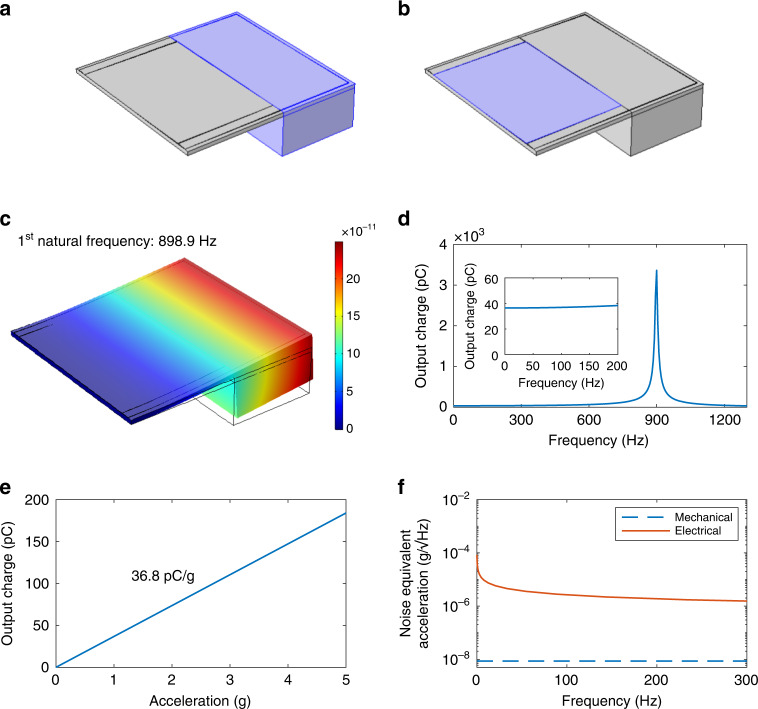
Modeled structure and simulation results of the designed accelerometer. **a**, **b** 3D structure modeled in COMSOL. Blue area in (**a**) is subject to inertial force. Blue area in (**b**) corresponds to the terminal for charge output. **c** The 1st vibration mode. The red indicates large displacement while the blue means smaller displacement. **d** Output charge versus frequency from 0.1 to 1300 Hz. Inserted is the enlarged view from 0.1 to 200 Hz. **e** Short-circuit output charge of the accelerometer versus excitation acceleration. **f** Thermal mechanical and thermal electrical noise equivalent acceleration of the accelerometer versus excitation frequency

We select structural health monitoring as the target application for our design, where the vibrations are usually small. Commercial products for this application generally have a working bandwidth from tens to hundreds of Hz and noise density from a few to tens of $\mu {{{\rm{g}}}}/\sqrt{{{{\rm{Hz}}}}}$^32–34^. Therefore, we set the working bandwidth higher than 100 Hz and noise density lower than 30 $\mu {{{\rm{g}}}}/\sqrt{{{{\rm{Hz}}}}}$ as our design targets. To verify the frequency range and noise level of the accelerometer design, we perform simulation via COMSOL Multiphysics software. 3D layout of the design is shown in Fig. 2a, b. The 0.22 μm Pt/Ti top electrode layer is neglected as its thickness is far less than that of piezoelectric and bottom electrode layers. The material properties of PZT, the Young’s modulus and Poisson’s ratio of stainless steel are the historical measurement data in laboratory. The density of stainless steel are provided by the supplier. The material properties of tungsten are obtained from COMSOL Material Laboratory. For the properties of PZT in strain-charge form, the compliance matrix is

1 $${s}^{E}=\left[\begin{array}{cccccc}13.6&-4.47&-5.86&0&0&0\\ -4.47&13.6&-5.86&0&0&0\\ -5.86&-5.86&16.9&0&0&0\\ 0&0&0&43&0&0\\ 0&0&0&0&43&0\\ 0&0&0&0&0&36.1\end{array}\right]\times 1{0}^{-12}\frac{1}{{{{\rm{Pa}}}}},$$

Permittivity matrix is

2 $${\varepsilon }^{T}=\left[\begin{array}{ccc}1160&0&0\\ 0&1160&0\\ 0&0&1010\end{array}\right]\times 8.854\,\times\, 1{0}^{-12}\frac{{{{\rm{F}}}}}{{{{\rm{m}}}}},$$

Piezoelectric constants are demonstrated in Table 1. For convenience, we represent the constants in a coupling matrix as shown in Eq. (3).

3 $$d=\left[\begin{array}{cccccc}0&0&0&0&125.5&0\\ 0&0&0&125.5&0&0\\ 17&17&73&0&0&0\\ \end{array}\right]\times 1{0}^{-12}\times \frac{{{{\rm{C}}}}}{{{{\rm{N}}}}},$$

**Table 1:** Piezoelectric constants of PZT thick film

Piezoelectric constants	*d*_31_	*d*_33_	*d*_15_
Value (×10^−12^ C/N)	17^48^	73	125.5

For stainless steel, the density is 7800 kg/m^3^, Young’s Modulus is 185 GPa, and Poisson’s ratio is 0.27. For tungsten, the density is 17800 kg/m^3^, Young’s Modulus is 360 GPa, and Poisson’s ratio is 0.27.

In solid mechanics, we select Rayleigh damping and use damping ratio *ζ* = 0.006. This value is obtained by solving *ζ* = 1/2*Q*^35^, where *Q* is the measured quality factor of the designed accelerometer in Table 2. As for boundary conditions, we set all the surfaces of the left side in Fig. 2a as fixed constraint. And for body load, a uniform harmonics force *F*_*V*_ = density *ρ* × 1g per unit volume is exerted on the proof mass as well as all the areas right on its top (blue area in Fig. 2a). In electrostatics, the bottom surface of the piezoelectric layer is set grounded (0 V). While the top surface of the piezoelectric layer where the top electrodes covers (blue area in Fig. 2b) is regarded as the terminal for charge output.

**Table 2:** The parameters for noise simulation of the accelerometer

Parameter	*T*	*f*_0_	*m*	*Q*	*η*	*C*	*Q*_*T*_
Value	297 K	867.4 Hz	0.15 g	84	0.03 @40 Hz	4.58 nF	22.74 pC/g

The 1st vibration mode of the designed structure is visualized in Fig. 2c. The 1st natural frequency corresponding to this mode is 898.9 Hz. Figure 2d demonstrates the frequency response of the 1-axis accelerometer at 1*g* gravitational acceleration from 0.1 to 1300 Hz. The inserted figure is the enlarged view of the frequency response from 0.1 to 200 Hz. In this range, the output charge remains less than 5% variation, which means that using this device to measure accelerations less than 200 Hz will give less than 5% error. The 200 Hz upper-frequency limit meets our design target for working bandwidth (>100 Hz). Figure 2e illustrates that the short-circuit output charge of the accelerometer has a linear relationship with the excitation acceleration at 95 Hz. Obtained by the slope of the line in this figure, the accelerometer has a charge sensitivity of 36.8 pC/g.

The noise of the designed piezoelectric accelerometer originates from mechanical-thermal noise (as the cantilever beam structure works as a mechanical harmonic oscillator) and the electrical–thermal noise of the piezoelectric material. The thermal mechanical and thermal electrical noise equivalent acceleration *a*_nm_, *a*_ne_ are represented by^36^:

4 $${a}_{nm}=\sqrt{\frac{4{k}_{B}T{\omega }_{0}}{mQ}},{a}_{\rm{ne}}=\sqrt{\frac{4{k}_{B}T\eta C}{\omega {Q}_{T}^{2}}}.$$

where *k*_*B*_ is the Boltzmann’s constant (1.38 × 10^−23^ J/K), *T* is the absolute temperature. The angular frequency *ω*_0_ = 2*πf*_0_, where *f*_0_ is the first resonance frequency. And *m* is the mass of the accelerometer, *Q* is the quality factor of the mechanical harmonic oscillator, *η* is the dissipation factor of the piezoelectric material, *C* is the electrical capacitance of the piezoelectric material. The angular frequency *ω* = 2*πf*, where *f* is the excitation frequency, *Q*_*T*_ is the charge sensitivity. *a*_nm_ is an invariant with respect to frequency when *f* < < *f*_0_. Taking the square root after adding the squares of *a*_nm_ and *a*_ne_, the total noise *a*_na_ of the sensor is calculated by the following equations^36^:

5 $${a}_{\rm{na}}=\sqrt{4{k}_{B}T\left(\frac{{\omega }_{0}}{mQ}+\frac{\eta C}{\omega {Q}_{T}^{2}}\right)}.$$

The detailed parameters for noise simulation are summarized in Table 2. *m* is estimated by multiplying the density of the tungsten by the volume of the proof mass. The experimental setup for other measured parameters is explained in the section Materials and Methods. Figure 2f shows the simulated results of *a*_nm_ and *a*_ne_ from 0.1 to 300 Hz. The thermal electrical noise *a*_ne_ is dominant over the thermal mechanical noise ${a}_{nm}(8.6\,{{{\rm{ng}}}}/\sqrt{{{{\rm{Hz}}}}})$. At 10, 20, and 95 Hz, the simulated total noise equivalent accelerations *a*_na_ are 8.3, 5.9, and 2.7 $\mu {{{\rm{g}}}}/\sqrt{{{{\rm{Hz}}}}}$.

### Experimental results

**Fig. 3:**
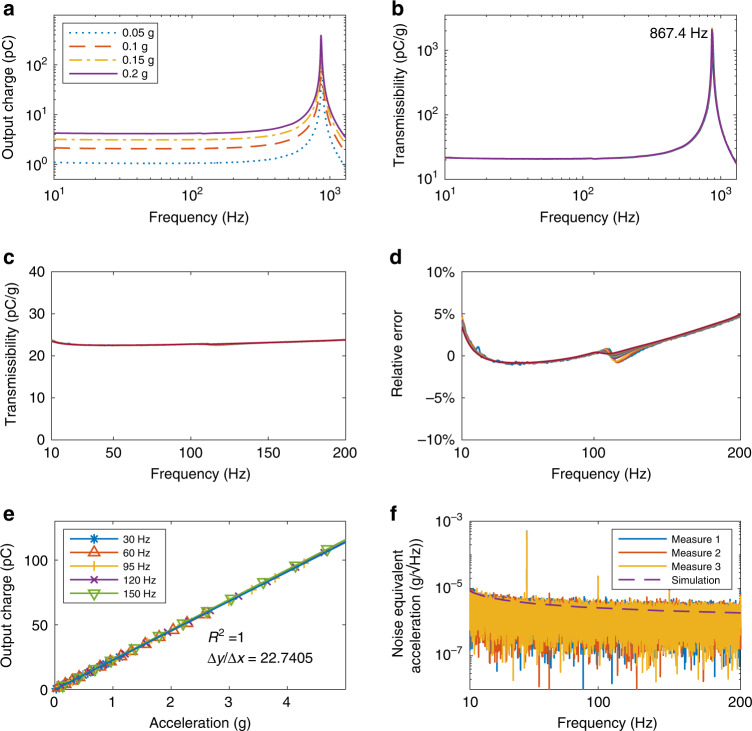
Experimental results. **a** Measured frequency response from 10 to 1300 Hz at 0.05, 0.1, 0.15, and 0.2 g. **b** Transmissibility of output charge to acceleration in (**a**). **c** Transmissibility of output charge to acceleration from 10 to 200 Hz. **d** Measurement error from 10 to 200 Hz (relative to the transmissibility at 95 Hz in (**c**)). **e** Relationship between excitation acceleration and output charge of the accelerometer from 0.05 to 5 g at different frequencies. **f** Measured noise equivalent acceleration from 10 to 300 Hz

Frequency response, sensitivity, and noise performance of the designed accelerometer are measured. Frequency responses from 10 to 1300 Hz at 0.05 to 2 g accelerations are demonstrated in Fig. 3a. It shows that the accelerometer has a flat response at low frequencies in all shown accelerations. Using the output charge divided by acceleration, we obtain transmissibility in Fig. 3b. The four curves coincide with each other with a degree of confidence of 95%. The relative error of the transmissibility at four peaks is 6%. At 0.1 g, the 1st resonance frequency of the accelerometer is 867.4 Hz.

To obtain the working bandwidth, we measure the frequency response of the accelerometer 14 times at different accelerations, as shown in Fig. 3c. We take the transmissibility at 95 Hz as a reference and representing *y*-axis in terms of relative error, giving Fig. 3d. For all 14 measurements, the lower and upper frequency limits that keep output charge in 5% relative error are around 10 and 200 Hz, respectively. This indicates that the working frequency of our designed accelerometer ranges from 10 to 200 Hz in 5% measurement inaccuracy.

To study the linearity and sensitivity of the accelerometer, we also extract the output charge *Q*_*a*_ at the accelerations from 0.05 to 5 g from the measured data in Fig. 3c. *Q*_*a*_ in different accelerations and frequencies are plotted in Fig. 3e. We get the charge sensitivity *Q*_*T*_ of 22.74 pC/g from the slope of the fitting line at 95 Hz. Linear regression gives *R*^2^ value of 1 for all frequencies, showing great linearity of the accelerometer.

As for noise performance, we measure the noise equivalent acceleration (NEA) from 10 to 200 Hz three times and present the results in Fig. 3f. The figure shows that NEA of the system is 7.7, 5.6, 3.6, 2.6 $\mu {{{\rm{g}}}}/\sqrt{{{{\rm{Hz}}}}}$ at 10, 20, 95, and 200 Hz. The minimum detectable acceleration of the designed accelerometer can be estimated by the peak-to-peak value of the noise. With a probability of 0.9973, we can assume that the peak-to-peak value of the noise is less than or equal to six times the RMS value. Using a single-pole filter to set the system bandwidth, the RMS value is estimated by^37^

6 $$\,{{\mbox{RMS Noise}}}={{\mbox{Noise density}}}\,\times \sqrt{{{{\rm{Bandwidth}}}}\times 1.6},$$

where the bandwidth of the accelerometer is 200 Hz in our case. Noise density specifies the noise density at the end frequency of the bandwidth, which is 2.6 $\mu {{{\rm{g}}}}/\sqrt{{{{\rm{Hz}}}}}$ at 200 Hz. The equation gives the RMS Noise value of 46.5 μg. Therefore, the peak-to-peak value of the noise is 279 μg, which means our designed accelerometer can detect an acceleration above 279 μg with a 99.7% degree of confidence.

Finally, specification of the designed accelerometer is summarized in Table 3.

**Table 3:** Specification summary of the designed accelerometer

Specification	Value	Measurement Times	Standard Deviation
Sensitivity	22.74 pC/g	5	0.25 pC/g
Frequency range	10–200 Hz (5%)	14	N/A
Natural frequency	867.4 Hz	4	4.39 Hz
Noise	5.6 $\mu {{{\rm{g}}}}/\sqrt{{{{\rm{Hz}}}}}$ at 20 Hz (with circuit)	3	0.0634 $\mu {{{\rm{g}}}}/\sqrt{{{{\rm{Hz}}}}}$

### Comparison

**Fig. 4:**
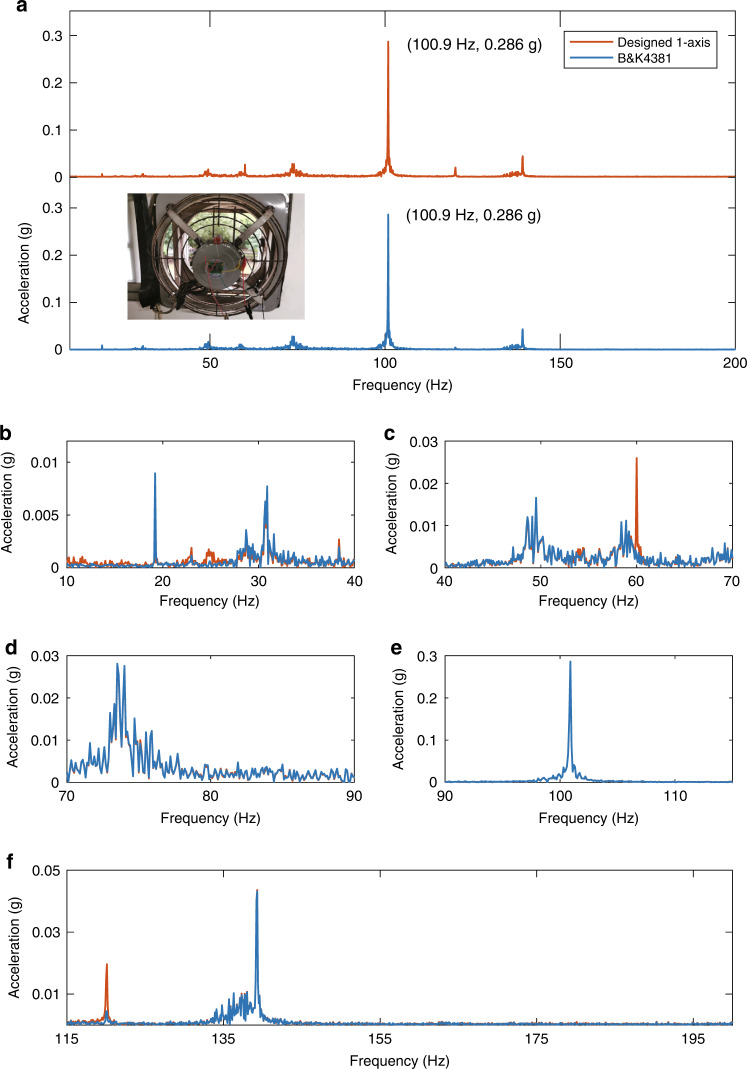
Fan vibration measurement with designed accelerometer and B&K4381. **a** Measured vibration signal of the fan by designed 1-axis accelerometer and B*&*K 4381 in frequency domain from 10 to 200 Hz. Inserted image shows the fan being measured and the two sensors placed on the fan. **b**–**f** Enlarged view of **a** from 10 to 40 Hz, 40 to 70 Hz, 70 to 90 Hz, 90 to 115 Hz and 115 to 200 Hz

To validate our accelerometer’s performance for actual application, we measure the vibrations of a fan with our accelerometer and a commercial accelerometer B*&*K 4381. As inserted in Fig. 4a, two accelerometers are bonded to top of a fan. We treat the sampled output signals of two accelerometers using Fast Fourier Transform (FFT) and get the frequency spectrum of the accelerations, as presented in Fig. 4a. Both accelerometers show that the dominant frequency and amplitude of the fan vibration are 100.9 Hz, 0.286 g. The enlarged views of Fig. 4a are plotted in Fig. 4b–f for a clearer comparison between the two results. The frequency spectrums of the two accelerometers match quite well, indicating the ability to accurately detect vibration with the use of our accelerometer. The spectra not matching well at around 25 Hz may come from the air flow. During fan vibration measurement, air flow blown by the fan blade passed through the two accelerometers. The flow could not influence the response of the sensing element of B&K 4381 attributed to its packaging. But our designed accelerometer might produce a response at 25 Hz under the flow since it was not packaged during testing. At 60 and 120 Hz, the designed accelerometer shows two peaks caused by 60 Hz power line interference because it is not shielded like B*&*K 4381.

**Fig. 5:**
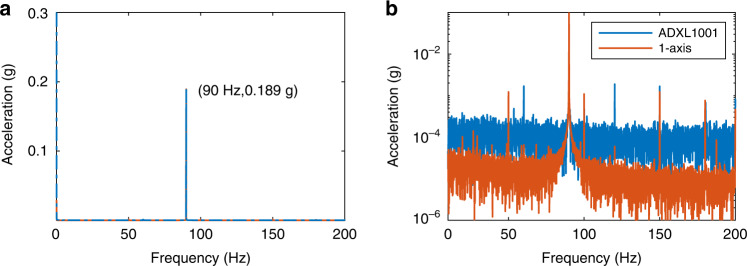
Noise performance of the designed accelerometer and ADXL1001. **a** Measured vibration signal of a shaker in frequency domain by the designed 1-axis accelerometer and ADXL1001. **b** Enlarged view of **a** after scaling *y*-axis

We also compare the noise performance of our designed accelerometer with that of a commercial accelerometer (ADXL1001). The two sensors are placed on a vibrating shaker, where 78 Hz, 0.881 g acceleration is measured by both sensors, as shown in Fig. 5a. To observe the noise level, we enlarge the *y*-axis of Fig. 5a and obtain Fig. 5b. It shows our designed accelerometer exhibits a noise floor of around 50 μg, much less than that of ADXL1001, about 300 μg.

## Discussion

In this section, the designed sensor is compared with other piezoelectric MEMS accelerometers to discuss the differences between their performances. It is also compared with commercial MEMS sensors to demonstrate its potential for low-noise applications.

Table 4 shows the comparison of reported piezoelectric MEMS accelerometers and our designed accelerometer in piezoelectric material, noise density, charge sensitivity, natural frequency, etc. For the papers not containing detailed dimension information, we estimate the volumes by multiplying the maximum length, maximum width, and maximum thickness of the active sensing element (excluding the fixed end) according to the given images of actual structures in these papers. And for the papers that have given the capacitances of the sensing elements, we also use them to calculate charge or voltage sensitivity.

**Table 4:** Summary of reported piezoelectric MEMS accelerometers in recent years

Research group		Material	Volume*	Noise density	Charge sensitivity	Voltage sensitivity	Natural frequency	Beam type
\		\	mm^3^	$\mu {{{\rm{g}}}}/\sqrt{{{{\rm{Hz}}}}}$	pC/g	mV/g	\	\
This work		PZT	20.97	5.6 @20Hz	22.74	4.96***	867.4 Hz	Cantilever
Trivedi et al.^24^		PZT	0.4	5800 @N/A	N/A	8.12	9.62 kHz	Four-symmetric
Hu et al.^43^		AlN	14.25	N/A	6.1***	7.95	1.29 kHz	Cantilever
Chen et al.^38^		AlN	32.4**	N/A	N/A	1.49	7.2 kHz	Ring
Tsai et al.^39^		PZT	33.92	N/A	N/A	2.21	6.5 kHz	Ring
Gesing et al.^23^		AlN	1.6	510 @100Hz	0.0981	N/A	19.1 kHz	Four-symmetric
Shen et al.^44^		PLZT	2.205**	N/A	0.04	N/A	7.68 kHz	Four-symmetric
Saayujya et al.^22^		ZnO	1	N/A	N/A	1.69	2.19 kHz	Cantilever
Hewa-Kasakarage et al.^17^	Beam 1	PZT	1.417**	N/A	5.1	3.26	363.95 Hz	Cantilever
	Beam 3	PZT	6.175**	1.74 @20Hz	50	15.67***	67.37 Hz	Cantilever
	Beam A5	PZT	0.975**	12.7@100Hz	3.43***	1	482.3 Hz	Cantilever

*Volume of the sensing element of the piezoelectric accelerometer

**Obtained by estimation according to the given picture of the real fabricated sensor in the corresponding paper

***Obtained using voltage/charge sensitivity multiplying/divided by the given capacitance of the piezoelectric sensing material

The designed sensor in our study exhibits a much higher charge sensitivity (22.74 pC/g) and a similar level voltage sensitivity (4.96 mV/g) compared to the sensors in most other studies. The main factors that influence the sensitivity of a piezoelectric accelerometer include structure shape, dimension of beams, seismic mass, piezoelectric constants, and capacitance of piezoelectric material^18^. As for structure shape, cantilever-beam structure results in higher sensitivity and lower natural frequency than symmetric-suspension beams^18^ or ring under the same overall size. This probably explains why our designed sensor shows higher voltage sensitivity (4.96 mV/g versus 1.49, 2.12 mV/g) even with a smaller sensing element volume (20.97 mm^3^ versus 33 mm^3^) compared to the sensors developed by Chen et al.^38^ and Tsai et al.^39^. When it comes to other factors, the generated charge of both the cantilever beam and four-symmetric beam is proportional to the mass, piezoelectric constant, and the square of beam length, but inversely proportional to the capacitance^40–42^. Hu et al.^43^ also chose the *d*_31_ mode sensing principle and cantilever beam structure. But the charge sensitivity of our designed sensor is much higher than that of the accelerometer in their study, even though the resonant frequencies and voltage sensitivities are similar to each other. We speculate the reason is that the piezoelectric coefficient *d*_31_ of our sensing material PZT is higher than that of AlN. Compared to the sensors fabricated by Gesing et al.^23^, Shen et al.^44^, and Saayujya et al.^22^, the sensor in this study has a longer beam and larger proof mass, which should be the primary reasons for the big differences in charge/voltage sensitivities and resonant frequencies. Compared to Beam 1 and Beam A5 developed by Hewa-Kasakarage et al.^17^, our fabricated sensor has both higher charge sensitivity (22.74 pC/g versus 5.1, 3.43 pC/g) and higher natural frequency (867.4 Hz versus 363.95 Hz, 483.2 Hz) probably due to much thicker piezoelectric film.

When it comes to noise density, our fabricated accelerometer shows a much lower noise level than accelerometers proposed by Trivedi et al.^24^, Gesing et al.^23^, and beam A5 of Hewa-Kasakarage et al.^17^. In Fig. 2f and Eq. (5), we have demonstrated that the dominant noise for the sensor in this study is thermal electrical noise *a*_ne_, which is proportional to $\sqrt{C}\eta /{Q}_{T}$. This implies that higher *Q*_*T*_ should be the main reason for the much lower noise density of our designed accelerometer.

Beam 3 developed by Hewa-Kasakarage et al.^17^ has a higher charge sensitivity *Q*_*T*_ (50 pC/g versus 22.74 pC/g) and a lower noise density *a*_*n*_ (1.74 $\mu {{{\rm{g}}}}/\sqrt{{{{\rm{Hz}}}}}$ at 20 Hz versus 5.6 $\mu {{{\rm{g}}}}/\sqrt{{{{\rm{Hz}}}}}$ at 20 Hz) but a lower resonant frequency *w*_*n*_ (67 Hz versus 867.4 Hz) than ours. It seems vague to compare Beam 3 and our designed accelerometer with these specifications, so we do an estimation to make the two devices comparable. As we know, higher charge sensitivity *Q*_*T*_ and higher resonant frequency *w*_*n*_ are desired when choosing an accelerometer, but these two parameters generally have a trade-off. For example, decreasing beam thickness *t* could improve the charge sensitivity *Q*_*T*_, but resonant frequency *w*_*n*_ would be lowered at the same time. Charge sensitivity *Q*_*T*_ and resonant frequency *w*_*n*_ are mainly influenced by thickness *t*, length *l*, and mass *M*. *Q*_*T*_ of a beam-based piezoelectric accelerometer is proportional to *M*(*l*/*t*)^2^^18^. According to the beam model^45^ and vibration theory^35^, *w*_*n*_ of an accelerometer is proportional to (*t*/*l*)^3/2^ × *M*^1/2^. Beam 3 has *l* = 8.5 mm, *t* = 20 μm, *m* = 0.771 mg, while for our accelerometer, *l* = 4.5 mm, *t* = 100 μm, *m* = 150 mg. Let us modify the size of our designed accelerometer to make it approximately equal to that of Beam 3, 6.175 mm^3^. We first reduce the thickness of the mass to 0.3 of original mass (0.3 mm) and the beam thickness to 0.3 of original thickness (33 μm), which means the size of our accelerometer will be 6.29 mm^3^. Then, *Q*_*T*_ would roughly be 0.3 × (1/(0.3))^2^ = 3.33 times, and *w*_*n*_ would approximately reduce to (0.3)^3/2^ × (0.3)^1/2^ = 0.09. This means our *Q*_*T*_ and *w*_*n*_ would be around 75.7 pC/g and 78 Hz. These results are better than the sensitivity and resonant frequency of Beam 3 proposed by Hewa-Kasakarage et al.^17^, which might be attributed to the thicker piezoelectric films achieved by aerosol deposition than that by sol–gel deposition.

Then, we consider the noise density *a*_*n*_ at 20 Hz. It is mainly determined by thermal electrical noise *a*_ne_, represented by Eq. (4) in our paper. The above-mentioned adjustment will only change *Q*_*T*_ among the parameters in Eq. (4). This means *a*_*n*_ is roughly proportional to 1/*Q*_*T*_, giving *a*_*n*_ = 1/3.33 × 5.6 $\mu {{{\rm{g}}}}/\sqrt{{{{\rm{Hz}}}}}$ = 1.68 $\mu {{{\rm{g}}}}/\sqrt{{{{\rm{Hz}}}}}$, which is comparable with 1.74 $\mu {{{\rm{g}}}}/\sqrt{{{{\rm{Hz}}}}}$ of Beam 3.

We also list some commercial low-noise MEMS accelerometers for comparison as shown in Table 5, including ADXL1001 that we have tested in Fig. 5. These accelerometers are suitable for low noise applications such as structural health monitoring, seismic imaging, condition monitoring, inertial navigation, etc. The designed sensor shows lower noise density than most of these accelerometers, which indicates that it is also suitable for some of these applications, such as bridge vibration mode detection^46^, imbalance and misalignment in fault detection^47^. For some cases with demand for wider measurement bandwidth, e.g., bearing and gears fault detection^47^, its frequency range can be further improved by adjusting the proof mass to meet the requirement.

**Table 5:** Summary of commercial low-noise MEMS accelerometers and designed sensor in this work

Model	Axis	Volume	Noise density	Sensitivity	Bandwidth	Dynamic range
\	\	mm^3^	$\mu {{{\rm{g}}}}/\sqrt{{{{\rm{Hz}}}}}$	mV/g	\	g
This work	1	20.97	5.6 @20 Hz	4.96(Not amplified)	5%, 200 Hz	N/A
LIS2LO6AL	2	40	30	480 to 1050	10%, 100 Hz	±2/± 6
LIS3L02AS4	3	264.328	50	160-240/480-720	10%, 100 Hz	±2/± 6
LIS344ALH	3	24	50	160-240/480-720	10%, 1800 Hz	±2/± 6
ADXL354	3	73.92	22.5 @1 Hz	400	3 dB, 1.9 kHz	±2
ADXL356	3	73.92	75 @1 Hz	80	3 dB, 2.4 kHz	±10
ADXL1001	1	45	40 @1 Hz	20	5%, 4.7 kHz	±100
ADXL1003	1	45	45 @100–10 kHz	10	5%, 6.2 kHz	±200
ADXL1005	1	45	75 @100–20 kHz	20	5%, 9 kHz	±100
SDI-1521J-005	1	250.56	12 @typical	800	5%, 400 Hz	±5
SI1003	1	259.2	0.7 @in band	900	3 dB, 550 Hz	±3
HP MEMS	1	72	0.02 @1 Hz	91,900	3 dB, 200 Hz	±0.08
MS1000T	1	255.85	102 @in band	90	3 dB, 200 Hz	±30

As for the applications with a higher requirement for noise performance, e.g., sub-$\mu {{{\rm{g}}}}/\sqrt{{{{\rm{Hz}}}}}$ noise level in seismic imaging^14^, the sensor in this study also shows great potential because the total noise density of the sensor could be reduced further by increasing charge sensitivity according to Eq. (5). Practical methods to increase charge sensitivity of the sensor include reducing the beam thickness, increasing beam length and proof mass. Beam thickness reduction can be achieved by adjusting the thickness of the substrate while keeping that of the piezoelectric film constant, e.g., changing the thickness of stainless steel from 100 to 30 μm. Proof mass adjustment was demonstrated in our previous work^27^, where the simulated noise of the structure with a larger proof mass (Structure 3) is around 3.2 $\mu {{{\rm{g}}}}/\sqrt{{{{\rm{Hz}}}}}$ at 20 Hz. Through Table 5, we could also observe that some capacitive MEMS accelerometers with a sub-$\mu {{{\rm{g}}}}/\sqrt{{{{\rm{Hz}}}}}$ noise floor have already been commercialized, such as SI1003 (0.7 $\mu {{{\rm{g}}}}/\sqrt{{{{\rm{Hz}}}}}$) and HP MEMS (0.02 $\mu {{{\rm{g}}}}/\sqrt{{{{\rm{Hz}}}}}$). Besides, a number of studies have strived for methods to reduce the noise floor from the direction of increasing proof mass, lowering spring constant, and increasing mechanical quality factor *Q*. However, as mentioned in the Introduction, these methods also lead to additional noise and higher power consumption due to the requirement for more complex circuits or the increase in cost and fabrication complexity because of vacuum packaging^14^. In comparison, piezoelectric sensing could achieve high *Q* directly without vacuum sealing, which potentially simplifies fabrication and lowers the cost.

## Materials and methods

### Fabrication

**Fig. 6:**
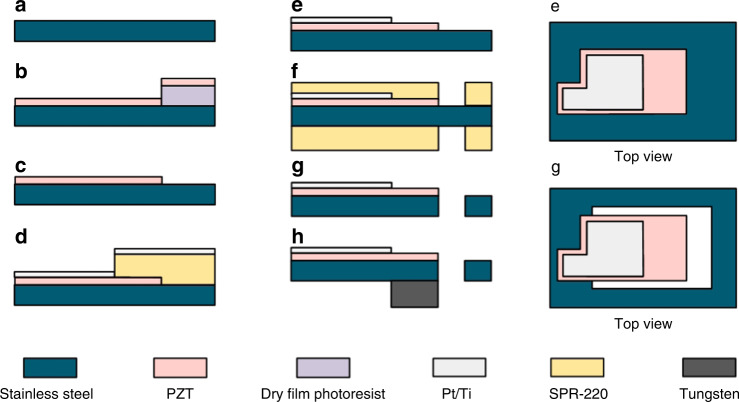
Schematic fabrication flow of the 1-axis piezoelectric accelerometer. **a** Clean substrate. **b** Deposit PZT thick film. **c** PZT lift-off. **d** Deposit Pt/Ti electrodes. **e** Pt/Ti lift-off. After lift-off, top view of the structure is shown on the right. **f** Define beam shape. **g** Release beam. Released structure from the top view is illustrated on the right. **h** Anneal and pole PZT, bond proof mass

We fabricate the accelerometer as presented in Fig. 6. Aerosol deposition method is chosen for high-quality hard PZT (PbZr_0.52_Ti_0.48_O_3_) thick film fabrication on the stainless steel substrate. The detailed piezoelectric properties of the PZT are introduced in Table 1. The fabrication starts from a piece of 100 μm thick 301 stainless steel (Fig. 6a), which is pre-cleaned by soaking in a 3:1 mixture of hydrogen peroxide (H_2_O_2_) and sulfuric acid (H_2_SO_4_) for 5 min to remove metal oxide and organic residues. After coating a layer of the negative dry-film photoresist THB-151N with photolithography, around 10 μm PZT layer is deposited via aerosol deposition (Fig. 6b). Followed by a lift-off process, a beam pattern is formed on the surface of the stainless steel substrate (Fig. 6c). After depositing SPR-220 with second photolithography, 20 nm titanium (Ti) and 200 nm platinum (Pt) layers are deposited using an electron beam evaporator (Fig. 6d). The shape of the top electrodes is defined via lift-off processes (Fig. 6e). Then, another photolithography process defines the wet-etching area (Fig. 6f). The beams are released after removing this area by aqua regia completely, and the photoresist SPR-220 is removed using acetone and isopropyl alcohol (IPA) (Fig. 6g). After fabrication in cleanroom, the beams are annealed in a furnace at 650 °C for 24 h to enhance the ferroelectric characteristics of the PZT layer. Finally, the PZT layer is poled at 120 V DC voltage and 423 K temperature for around 2 h, and the beam is bonded with the proof masses using CA40H glue (Fig. 6h).

**Fig. 7:**
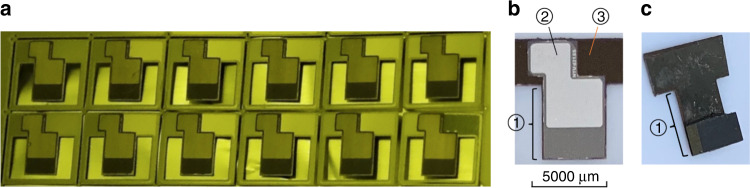
Fabricated sensors. **a** A batch of fabricated sensors before annealing on a piece of aluminum foil in yellow light room. **b**, **c** Top and bottom views of a fabricated accelerometer with proof mass

The process was first proposed to fabricate a piezoelectric energy harvester by Lin et al.^48^. Compared to the silicon substrate, stainless steel results in higher capacitance and open-circuit output voltage, which enables larger charge sensitivity. Besides, the higher fracture toughness is beneficial to the working lifetime, especially when the structure is excited by large acceleration^49^. Figure 7 shows the fabricated sensors before annealing (a) and after poling (b and c). Part ① in Fig. 7b corresponds to the sensing structure as shown in Fig. 1. Parts ② and ③ act as the plates for poling and connecting the probe lines during testing.

### Experimental setup

**Fig. 8:**
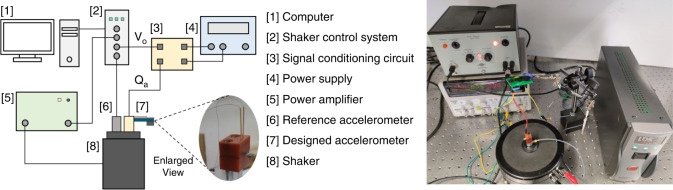
Experimental setup for frequency response measurement. The left figure shows the schematic diagram of the testing setup in the right photo

The frequency response is measured with the experimental setup as shown in Fig. 8. A shaker control system (B&K LDS-Dactron) is used to control the whole frequency sweep process. The signal generated by the control system is amplified by a power amplifier (B&K Type 2706) and excited a shaker (B&K 4809) to produce vibration. The designed accelerometer and a commercial accelerometer (B*&*K 4513) are clamped by a fixture and fixed to a shaker (B*&*K 4809). The output charge of the designed accelerometer (shown in the enlarged view) is first amplified by a self-designed signal conditioning circuit with the gain *G*_*c*_ = 4.43 mV/pC. Then, the amplified signal is sampled by the shaker control system. The shaker’s acceleration measured by B*&*K 4513 is regarded as the reference signal, which is also sampled by the shaker control system.

The noise performance is obtained by measuring the voltage noise spectral density of the testing system (designed accelerometer connected with the designed charge amplifier) by PHOTON+ Dynamic Signal Analyzer. Then, we could obtain the noise equivalent acceleration using voltage noise spectral density divided by the total sensitivity of the testing system *S*_sys_ = *G*_c_*Q_*T*_ = 100.4 mV/g.

For the data in Table 2, the temperature *T* is measured using a thermometer. *f*_0_ and *Q*_*T*_ are obtained from Fig. 3b and e, respectively. Impedance Analyzer Agilent 4294A is employed to get the dissipation factor *η*. Capacitance *C* of the PZT layer is measured via LCR meter Keysight U1733C at room temperature *T*. *Q* is derived using the resonant frequency *f*_0_ divided by the half-power bandwidth Δ*f* obtained in Fig. 3b.

## Conclusion

A 1-axis cantilever beam micro piezoelectric accelerometer is developed in this study. To increase sensitivity, aerosol deposition is applied to form thick PZT sensing films in the MEMS fabrication process. Simulation is first performed to validate that the designed structure has suitable working bandwidth and noise level for our target application—structural health monitoring. Then, performance measurement shows that the accelerometer has a charge sensitivity of 22.74 pC/g, natural frequency of 867.4 Hz, working bandwidth of 10–200 Hz (within ±5% deviation), and noise equivalent acceleration of 5.6 $\mu {{{\rm{g}}}}/\sqrt{{{{\rm{Hz}}}}}$ (together with the signal conditioning circuit). Our sensor matches the commercial piezoelectric accelerometer B&K 4381 well in fan vibration measurement, demonstrating its ability to operate in real applications. Besides, a comparison with commercial capacitive MEMS accelerometer ADXL1001 shows that it has a much lower noise level. Finally, we compare the differences in performances of our sensor and other piezoelectric MEMS accelerometers. We also demonstrate that the sensor has great potential for low-noise applications compared to low-noise capacitive MEMS accelerometers.
